# AI at your service: AI tools for solving crystallographic problems

**DOI:** 10.1063/4.0001095

**Published:** 2025-10-27

**Authors:** Simon J. L. Billinge

**Affiliations:** Columbia University, New York, NY, USA

## Abstract

At the heart of powder diffraction is the idea that we are studying real materials doing real things, often in real devices. It is now possible to solve single crystal structures from tiny crystals that are smaller than powder grains so if powder diffraction was just for solving structures, we wouldn't need powder diffraction any more. But the field is as strong as ever, and if anything, getting stronger and more impactful, exactly because it is solving scientific and technological problems in real situations. However, this presents a number of key data analysis and interpretation challenges because it implies we are studying ever more complicated samples, often in complex heterogeneous devices and in time-resolved operando setups, and we are interrogating our data for more and more subtle effects such as microstructures and evolving defects and local structures. Advanced data analysis algorithms and software are essential for the success of this enterprise. In this talk I will describe various developments that leverage the power of artificial intelligence (AI), principally machine learning (ML), to aid in this task. Some of these powerful tools are clearly ready to be applied more broadly in the community and others are still in the future but look very promising. They include unsupervised and supervised machine learning approaches, conventional ML and deep neural networks, as well as approaches for autonomous time-resolved experimentation.

